# Appendicitis as a Cause of Intestinal Obstruction
1Read before a Meeting of the Bristol Medico-Chirurgical Society on November 12th, 1924.


**Published:** 1925-01

**Authors:** Richard Warren

**Affiliations:** Surgeon to the Weston-super-Mare Hospital


### APPENDICITIS AS A CAUSE OF INTESTINAL OBSTRUCTION.[1]

BY

RICHARD WARREN, M.D., M.Ch. Oxon., F.R.C.S.,

*Surgeon to the Weston-super-Mare Hospital.*

---

Few things in surgery are more remarkable than the behaviour of plastic lymph poured out by the peritoneum as a response to irritation. Though usually beneficial by closing perforations, or by walling off inflamed areas and so preventing widespread contamination, or by securing our crude surgical suturing of hollow viscera, yet occasionally the results are disastrous, as the lymph, even before organisation has properly occurred, may form bands or membranes which obstruct or kink the intestine and produce the unfortunate complication of intestinal obstruction.

Intestinal obstruction as a sequel to or a concomitant of appendicitis is fortunately not common, but on the other hand it is not extremely rare. The complication occurred 34 times in 1,011 cases of operation for appendicitis of which I have notes.

In addition there occurred in a certain proportion of cases a condition of loculated fibrino-purulent peritonitis associated with a certain amount of obstruction in which the lethal result was perhaps more from peritonitis than obstruction, and these cases have a very high mortality.

Until I began to look up the notes of cases I never appreciated the frequency and importance of appendicitis

[1] Read before a Meeting of the Bristol Medico-Chirurgical Society on November 12th, 1924.

as a cause of intestinal obstruction. Of 180 cases of obstruction upon which I have operated 68 were due to stricture (usually malignant), 54 were bands and kinks, 40 were intussusception; these are the big groups, the rest were volvulus 11, internal hernia 5 and gallstone obstruction 2. Now of the group of bands and kinks no less than 34 were definitely due to appendicitis, which makes it in my experience by far the most common cause of this form of obstruction. It is probably more common than these figures suggest, for these cases occurred either during or shortly after an attack of appendicitis, usually days or weeks, the longest no more than three months later. But in several cases of obstruction by bands the cause of the bands was not discovered at operation, and it is probable that a certain number of these were also the results of appendicitis.

*Clinically,* cases of obstruction due to appendicitis fall into two groups: (1) *Primary,* and (2) *Post-operative.* In the former group there were 13 cases, in the latter 21.

1. *Primary Obstruction.*—As a rule the whole affair is recent and acute, the symptoms suggesting acute obstruction, but possibly with fever and some localised tenderness and resistance in the right iliac fossa, suggesting something more than a simple obstruction. Of this type the following is a fairly typical example:—

A female, aged 70, ill twenty-four hours with repeated vomiting, abdominal distension and constipation. The temperature was subnormal, the pulse normal and there was tenderness in the right iliac fossa. Operation was performed immediately and showed an acutely inflamed appendix adherent to and kinking the lower ileum with marked distension of the gut above and emptiness of that below. Removal of the appendix from its lymph bed automatically released the gut and the patient made a good recovery. It was remarkable what a small amount of recent lymph, only a few hours or days old, could produce intestinal obstruction.

A similar clinical picture may be produced by obstruction of the lower colon with distension of the cæcum, which is becoming ulcerated and beginning to leak, and in a middle-aged or elderly patient the distinction between these two conditions is almost impossible.

A less common type is the following :—

A boy, aged 8, suffered with belly-ache and occasional vomiting for a month. His appetite was bad and he sweated at night. For two days before I saw him his bowels were not open and the vomiting was more urgent. When seen he had a typical abdominal facies, marked distension of the abdomen with visible peristalsis of small gut. The temperature was 100° F. and the pulse 100.

*Operation.*—Laparotomy was done immediately, and it was found that two or three feet of the ileum immediately above the cæcum were kinked and matted by adhesions, the ileum above was much distended and the cæcum and colon collapsed. An acutely inflamed appendix was removed from the middle of the matted ileum and the adhesions were broken down. As it was uncertain whether the intestinal contents would pass through such a length of injured intestine, a lateral anastomosis was made between the ileum above the kinked part and the cæcum, and also a valvular cæcostomy with No. 12 catheter was performed. The patient made a good recovery.

The mortality for 13 cases was nil.

2. *Secondary or Post-operative Obstruction.*—This form of obstruction was more common and far more fatal than the primary form, partly because of the difficulty of diagnosis, for it is not easy in the early stages to distinguish atonic ileus or early generalised peritonitis from obstruction by a recent band, partly because the patient has been ill longer and has already had one operation, so that vitality is more depressed. Of 21 cases in this group 7 died, *i.e.* about 30 per cent., which is the average mortality for intestinal obstruction in my experience.

Post-operative obstruction usually arises within a week of the primary operation with paroxysmal abdominal pain, vomiting which becomes green and later brown and possibly a subnormal temperature. The latter is very suggestive when it occurs, but is likely to be masked by the irregular fever of an accompanying peritonitis. Less commonly the obstruction develops later, weeks or months after an attack of appendicitis, with well-developed fibrous adhesions and bands arising from the former plastic lymph.. No doubt some of the bands which one divides in a case of acute obstruction are of appendical origin, but not proven as such, and not therefore included in this series. The following is an example of a late case of intestinal obstruction definitely appendicular in origin :—

A male, aged 42, was operated on for suppurating appendicitis three months ago, the appendix was removed and the abscess drained. Attacks of abdominal pain with distension and vomiting followed. These were relieved by enemata, but the attacks were recurring and becoming worse.

On examination there was a well-marked incisional hernia, a tense and distended abdomen, a pulse of 100 and an anxious expression.

Operation was undertaken at once.

Laparotomy was done through the previous scar in the right iliac fossa. The lowest foot of the ileum was badly kinked up by adhesions, the gut above being red and distended, the colon empty and contracted. After freeing the adhesions the gut was raw over an extensive area, and it seemed probable that kinking might recur, so an ileo-cæcostomy was done to short-circuit the kinked part of the ileum, the ventral hernia was repaired in layers with overlapping. He made a clean recovery. Later he developed acute cholecystitis.

*Diagnosis.*—Complete diagnosis may not be easy, but is less important in the primary cases, since in these it is usually pretty clear that the condition is one of "acute abdomen," with signs pointing partly to obstruction and partly to some inflammatory affection and that operation is urgently indicated. The only noteworthy point is to open the abdomen by the paramedian incision rather than in the right iliac fossa, so as to give plenty of room if adhesions have to be dealt with or an anastomosis performed.

In the post-operative type diagnosis may be difficult and is extremely important, as on the one hand one wants to relieve a true mechanical obstruction as soon as possible by operation, on the other one does not want to further handle and abuse inflamed intestine when the condition is really one of peritonitis and ileus.

A sudden attack of paroxysmal pain with rising pulse and falling temperature, occurring in a patient who has been going on smoothly after the first operation, is fairly suggestive of obstruction when associated with bilious vomit becoming brown, with a soft abdomen, and in such cases there should be no delay in opening the abdomen. But where the condition is a mixture of obstruction with suppurative peritonitis diagnosis and treatment are alike difficult.

The following is a good example of this type of case :—

A girl, aged 15, with a history of two days. At the first operation a gangrenous appendix was removed through a gridiron incision, and as it was surrounded by foul pus the wound was drained for two days. The patient did well for some days, then began to have irregular fever and per rectum signs of a pelvic collection. This was opened by a medium incision on the thirteenth day and proved to be of some size. It was drained with a large tube. Irregular pyrexia persisted, and a fæcal fistula developed. The condition was not satisfactory, but there was no evidence of loculated pus. Twenty days later, *i.e.* thirty-three days from the first operation the patient had severe abdominal pain and vomited eight times in the night, the temperature became subnormal and the face markedly abdominal.

A third operation was done, a higher mid-line incision being used, and many adhesions were broken down and a very small loculated abscess found and drained. It did not appear to be a very satisfactory operation, but the patient slowly recovered and was playing hockey three or four months afterwards.

This seemed to be mainly a case of fibrino-purulent peritonitis which incidentally developed obstruction.

*Treatment.*—In the cases quoted I have already referred to several of the methods possible. In most instances it is fairly simple dividing a band or bands of recent lymph, which can be done with the finger; only in the types of later onset do the adhesions require cutting. More important is the question of further treatment, such as drainage or anastomosis. When the gut above the obstruction is very distended and the contents do not readily flow past the seat of obstruction after its division the gut should be tapped with a medium trocar and gas and liquid fæces let out and the puncture closed with purse-string suture. If the condition of the gut still appears doubtful a temporary fæcal fistula should be made, and I have found an ileostomy of the self-closing type quite satisfactory, using a No. 12 catheter as a drain.

Where a large amount of small gut is matted, and it is doubtful if it will not rapidly become obstructed again, a short circuit should be done, usually an ileo-cæcostomy to which a valvular cæcostomy may with advantage be added in the more acute cases, as suggested by Handley some years ago for the condition he calls ileus duplex. I am doubtful of the occurrence of this condition as he describes it, viz. a block of the ileum and sigmoid at the same time. The ileus is, however, certainly duplicated in many cases in another sense, I mean that there is peritonitic as well as obstructive ileus. The effect of an ileo-cæcostomy with a cæcostomy is very similar to that of a simple ileostomy, and possibly better where the patient will stand this further manipulation.

Since I wrote the above an interesting paper on the same subject has appeared by Rayner of Manchester,[1] in which he mentions cases very similar to those I have described, and his conclusions are very similar to mine, except that in the treatment of post-operative obstruction he advises the use of stomach tube and morphia for one or two days before proceeding to operation and quotes a successful case. Personally I think that twelve hours is long enough to persist in such cases unless marked improvement is noted, and should think that ileus relieved by such treatment is more probably peritonitic than true obstruction.

Another paper bearing on this subject was from America. I have lost the reference. The author of this found that tube drainage in the right iliac fossa was followed far less by post-operative obstruction than where the drain was in the mid-line. I have not enough data to criticise this statement. I have usually employed the gridiron incision and all the post-operative cases have been drained, usually with the drain two days in the abdomen and a few more days in the abdominal wall, *i.e.* all the post-operative cases followed a fairly severe peritoneal infection whether localised or not.

### DISCUSSION.

The PRESIDENT thanked Mr. Warren for his interesting and valuable contribution, on which discussion was invited. As a physician he would be glad to hear whether Mr. Warren could recommend any measures that tended to prevent these post-operative obstructions. He recalled the late Mr. R. Poole Lansdown's advocacy of abdominal massage as soon as practicable after operation for appendicitis with a view to preventing the occurrence of adhesions.

Mr. RENDLE SHORT remarked that these cases of intestinal obstruction following appendicitis were often difficult to diagnose from peritonitis and from ileus, and that visible peristalsis was the most conclusive sign. In cases of ileus, when all other means of obtaining an action of the bowels had failed, he had several times seen lives saved by the

"triple attack"; this consists of giving a dose of two minims of croton oil, then four or five hours later a turpentine enema, and whilst it is still in the bowel a hypodermic of pituitary extract. Of course, this will not avail if there is an authentic obstruction. Under those circumstances ileostomy under novocain anæsthesia may be safer.

Mr. Ferrier Walters pointed out that these post-operative obstructive adhesions were not seldom difficult to diagnose, and that he regarded vomit becoming fæculent, after an interval of 48 to 72 hours free from this symptom, as suggestive of inflammatory obstruction. He preferred the pelvic drain, and had given up draining from the wound. It was important to take care that no coil of intestine was allowed to intervene between the drainage tube and the bladder. He would like to hear from Mr. Warren how often he had met with cases of Sampson Handley's ileus duplex. As to the incision, he invariably used Battle's incision, on females, where a doubt existed as to diagnosis, since this incision lends itself to such other operations as might prove necessary in cases in which the diagnosis proved incorrect. He used the gridiron, however, in men and children, as there is little liability to subsequent hernia, and one could get the patient up within a week.

Mr. Chitty, referring to the suggestion that obstruction was more often seen when Battle's incision was employed than when McBurney's operation was performed, said that obstruction was more common when pelvic drainage was necessary, and he considered that approaching the appendix from the inner side, as in a Battle's incision, had been more often followed by obstruction in his own cases than when he had adopted the gridiron incision, and so approaching the appendix more laterally. Consequently he preferred the latter incision unless the presence of pelvic suppuration was suggested by rectal examination, or unless some doubt as to the diagnosis renders a more extensive exploratory incision advisable.

Mr. TASKER said that he had used Battle's incision in over two hundred appendicectomies, and had only encountered two cases of post-operative obstruction. He had come to the conclusion that if a drainage tube had to be used, post-operative hernia more often followed McBurney's incision than if Battle's incision was used.

Mr. LACY FIRTH stated that after using the gridiron incision for many years he was led to adopt Battle's incision, which had the advantages in his opinion of giving a better exposure and of being easily extended for further investigation when the diagnosis was doubtful. He had not met with the cases of mechanical obstruction in acute appendicitis which Mr. Warren had described. The obstruction cases he had met with had been considered to be due to spreading peritonitis and treated as such.

Mr. RICHARD WARREN, in reply to points raised during the discussion by various speakers, said that he believed it was difficult to attribute the prevention of adhesions to any method adopted to that end, and he doubted whether massage of the abdomen avails or makes a material difference, although early movements of the patient while still confined to bed might be helpful. Mr. Short had referred to visible peristalsis soon after operation as a valuable diagnostic sign, but he had found visible peristalsis, at any rate in acute cases, was somewhat late in appearing, and if present he would have no hesitation in opening up the abdomen at once. The "triple attack" was too prolonged in action for an acute case which requires quick operation, but for the less acute the method might prove of value, though he felt a decided objection to employing croton oil. Mr. Warren said he had not encountered ileus duplex, and though he had cited Sampson Handley in this connection, he did not agree with him. Nevertheless, the method seems to have worked well. In ileo-cæcostomy with the tube in the cæcum one obtained direct rapid drainage of fæces from the ileum through the cæcum. He agreed that inflammation of a retro-cæcal appendix is more apt to be followed by adhesions than that of the more usually located appendix.

